# Dose variations in tumor volumes and organs at risk during IMRT for head‐and‐neck cancer

**DOI:** 10.1120/jacmp.v13i6.3723

**Published:** 2012-11-08

**Authors:** Mercè Beltran, Mónica Ramos, Juan José Rovira, Santiago Perez‐Hoyos, Marc Sancho, Enrique Puertas, Sergi Benavente, Mercè Ginjaume, Jordi Giralt

**Affiliations:** ^1^ Servei de Física Hospital Universitari Vall d'Hebron Universitat Autònoma de Barcelona Barcelona 08035 Spain; ^2^ Servei d'Oncologia Radioteràpica Hospital Universitari Vall d'Hebron Universitat Autònoma de Barcelona Barcelona 08035 Spain; ^3^ Servei de Medicina Preventiva i Salut Pública Hospital Universitari Vall d'Hebron Universitat Autònoma de Barcelona Barcelona 08035 Spain; ^4^ Institut de Tècniques Energètiques Universitat Politècnica de Catalunya Campus Diagonal Sud Edifici PC Barcelona 08028 Spain

**Keywords:** head‐and‐neck cancer, IMRT, dose‐volume changes, organs at risk

## Abstract

Many head‐and‐neck cancer (HNC) patients treated with radiotherapy suffer significant anatomical changes due to tumor shrinkage or weight loss. The purpose of this study was to assess dose changes over target volumes and organs at risk during intensity‐modulated radiotherapy for HNC patients. Sixteen HNC IMRT patients, all requiring bilateral neck irradiation, were enrolled in the study. A ${\text{CT}}_{\text{plan}}$ was performed and the initial dose distribution was calculated. During the treatment, two subsequent CTs at the 15th $\left({\text{CT}}_{15}\right)$ and 25th $\left({\text{CT}}_{25}\right)$ fractions were acquired. The initial plan was calculated on the ${\text{CT}}_{15}$ and ${\text{CT}}_{25}$, and dose‐volume differences related to the ${\text{CT}}_{\text{plan}}$ were assessed. For target volumes, mean values of nearmaximun absorbed dose ($D_{2\%}$) increased at the 25th fraction, and doses covering 95% and 98% of volume decreased significantly at the 15th fraction. Contralateral and ipsilateral parotid gland mean doses increased by 6.1% (range: ‐5.4, 23.5%) and 4.7% (range: ‐9.1, 22.3%), respectively, at ${\text{CT}}_{25}$. The $D_{2\%}$ in the spinal cord increased by 1.8 Gy at ${\text{CT}}_{15}$. Mean absorbed dose increases at ${\text{CT}}_{15}$ and ${\text{CT}}_{25}$ were observed in: the lips, 3.8% and 5.3%; the oral cavity, 3.5% and 2.5%; and lower middle neck structure, 1.9% and 1.6%. Anatomical changes during treatment of HNC patients affect dose distribution and induce a loss of dose coverage to target volumes and an overdosage to critical structures. Appropriate organs at risk have to be contoured and monitored in order to know if the initial plan remains suitable during the course of the treatment. Reported dosimetric data can help to identify patients who could benefit from adaptive radiotherapy.

PACS numbers: 87.53.Kn, 87.55.Dk

## I. INTRODUCTION

The benefit of intensity‐modulated radiation therapy (IMRT) in the treatment of head‐and‐neck cancer (HNC) has been demonstrated in numerous studies.^1^, ^3^ Highly conformal radiation allows for a high dose to high‐risk areas, whilst sparing adjacent organs at risk (OAR) such as the parotid glands. Clinical studies have shown that IMRT reduces grade‐3 xerostomia in comparison to three‐dimensional conformal radiotherapy (3D CRT).^4^, ^5^ For that reason, IMRT has become the standard treatment in many centers. IMRT dose distributions, with steep dose gradients, are very sensitive to geometrical uncertainties, and hence, deviations between planned and delivered dose distributions have to be minimized. One way of improving the treatment accuracy is to reduce geometrical errors. Rigid errors, such as setup, have been extensively studied. Mechalakos et al.^(6)^ for instance evaluated the interfraction and intrafraction errors in treatments of HNC and compared their results with previous studies from others authors. Margins are added to clinical volumes in order to take into account geometrical uncertainties. These planning margins are commonly calculated from measured systematic and random geometrical errors.^(7)^

However, it is well known that many HNC patients treated with radiotherapy (RT) suffer significant anatomical changes due to tumor shrinkage or weight loss. Several scheduled rescanning studies have evaluated these volumetric changes in both target volumes and normal tissues,^8^, ^11^ mostly on the parotid glands and their consequent effects on dose distribution.^12^, ^15^ The information obtained from these studies indicates that anatomical changes during the treatment can cause deviations between the planned and delivered dose, specifically reducing dosage to target volumes whilst increasing dosage to critical structures.

Adaptive RT based on replanning during treatment is a common strategy to minimize the effect of anatomical changes over dose distribution,^16^, ^17^ although identifying which patients in particular could best benefit from replanning is, as yet, undetermined. Ahn et al.^(18)^ indicated the need for an accurate determination of anatomical changes and their consequent dosimetric parameters as a prime factor in the determination of any subsequent replanning.

The purpose of the present study was to analyze volume variation repercussions on the dose distribution in both PTVs and organs at risk. The use of IMRT implies the irradiation of more OARs than conventional 3D CRT. Therefore, beside typical susceptible organs such as the eyes, spinal cord, parotid glands, and mandible, we have also included additional OARs such as the oral cavity, cochleae, lips, brain, brainstem, and lower middle neck structures. The influence of anatomical changes over dose distribution on these structures has not, until now, been reported in previous studies. The study was experimental and patients were treated without replanning.

## II. MATERIALS AND METHODS

### A. Eligibility criteria

Patients were required to have a histologically proven diagnosis of HN squamous cell carcinoma of the head and neck, staged T3‐T4 N0 or T1‐T4 N1‐N3. Patients were aged 18 years or over, with a Karnofsky performance score of ≤80%. Eligibility criteria also included bilateral neck irradiation. The protocol was approved by the local Ethics Committee. All enrolled patients signed an informed consent form.

### B. CT acquisition and contouring

CT scans were made using a LightSpeed RT 4s helical CT model (GE, Tokyo, Japan) with 2.5–5 mm slice spacing. Patients were in the supine position and immobilized with a thermoplastic head–shoulder mask accommodated on a VersaBoard immobilizer device (JRT Associates, Elmsford, NY). A planning CT scan $\left({\text{CT}}_{\mathrm{plan}}\right)$ was acquired one week before RT treatment. Second and third CT scans were performed during the course of treatment at the 15th $\left({\text{CT}}_{15}\right)$ and 25th $\left({\text{CT}}_{25}\right)$ fractions. Patient weight was recorded before treatment and at weekly intervals during therapy.

The Eclipse version 7.5 (Varian Oncology Systems, Palo Alto, CA) treatment planning system was used for delineation and dose distribution calculations. Target volumes and normal tissues were manually contoured by a physician on each axial slice of the ${\text{CT}}_{\text{plan}}$ using MRI or contrast‐enhanced CT. The definition of volumes was in accordance with ICRU Reports 50–62,^19^, ^20^ but dose‐volume parameters were reported according to the new ICRU Report 83^(21)^ IMRT recommendations. Gross tumor volume (GTV) included the primary tumor and affected lymph nodes. The GTV was expanded to include the high‐risk regions (CTV2) and the low risk nodal regions (CTV1). To compensate for geometrical uncertainties such as setup and organ motion, a 5 mm margin was automatically added to CTVs to obtain the planning target volume (PTV). In order to avoid dose compensation in the build‐up region, in cases with no skin infiltration, the PTVs were manually modified excluding areas where the distance to the skin was less than 3 mm. Although these modified PTVs were used during optimization process, the absorbed dose was reported over the whole PTV. Prescribed doses were between 51–54 Gy for PTV1 and 66–70 Gy for PTV2.

The critical structures contoured were: the parotid glands, spinal cord, mandible, eyes, oral cavity, brainstem, lips, cochleae, brain, and lower middle neck structures comprising the pharyngeal constrictors, glottic‐supraglottic larynx, and the esophagus. To avoid conflict between planning aims when OARs overlapped with PTVs, the former OARs were then automatically recontoured, excluding the region within PTV. The modified OARs were used in the optimization process, but the final OAR dose goals were evaluated in the entire OAR.

A limited external volume structure was contoured, representing the volume inside the external body contoured from the upper level of the PTV1 to the upper neck treatment area, thus avoiding the presence of the shoulders. This volume was added with the intention to correlate anatomical changes inside the treatment area with any weight change.

### C. Treatment planning

IMRT treatment plan was generated on the ${\text{CT}}_{\text{plan}}$ with seven or nine 6 MV fields, using the simultaneous integrated boost (SIB) technique, treating the entire neck with IMRT but avoiding matching fields.^(22)^ The IMRT plans were optimized using an inverse planning algorithm, namely Helios 8005 (Varian Oncology Systems). The final dose distribution was calculated using the Pencil Beam Convolution 8005 with heterogeneity correction and 5 mm grid resolution. Optimization goals were as follows: 1) prescription doses $\left(D_{\mathrm{pres}}\right)$ must encompass at least 95% of target volumes; 2) near‐minimum absorbed doses ($D_{98\%}$) of PTVs should be higher than 93% of $D_{\text{pres}}$; 3) the near‐maximum absorbed dose ($D_{2\%}$) of the PTVs should be less than 115% of $D_{\text{pres}}$; 4) PTV2 should be considered as the highest priority target volume.

High priority constraints to normal critical structures were: no more than $1.0\;{\text{cm}}^{3}$ of spinal cord could receive more than 48 Gy; 2) no more than 1% of brainstem could receive more 54 Gy; 3) $D_{2\%}$ for mandible should be less than 70 Gy; 4) the parotid gland volume receiving 26 Gy should be less than 50% in at least one gland; 5) $D_{2\%}$ of normal tissue should be less than $D_{\text{pres}}$.

Low priority constraints that should not compromise target coverage were: 1) the mean absorbed dose $\left(D_{\mathrm{mean}}\right)$ covering lower middle neck, oral cavity, and lips should be less than 40 Gy; 2) eyes $D_{2\%}$ should be less than 50 Gy; 3) cochleae $D_{2\%}$ should be less than $D_{\text{pres}}$.

All patients were treated with a Clinac 2100 accelerator (Varian Oncology Systems) with Millenium MLC‐80 leaves of 1 cm width in a Dynamic mode. In order to reduce systematic setup errors, portal images were acquired periodically, and corrections were made using a weekly offline protocol.

### D. Manual adaptive contouring

A simplified methodology was designed to evaluate volume variation during treatment with no attempt to modify the initial PTVs. ${\text{CT}}_{15}$ and ${\text{CT}}_{25}$ were registered using the initial reference marks and bony anatomy.

All contoured structures of ${\text{CT}}_{\text{plan}}$ were copied on the ${\text{CT}}_{15}$ and ${\text{CT}}_{25}$. Following this, all contours were manually adapted for any anatomical changes.

For PTVs, efforts were made to maintain the original clinical volumes, and were modified only to be consistent with the new anatomy — for instance, in cases where PTV extended beyond the skin or overlap areas limited by bones such as mandible and vertebra. We are well aware that PTV changes during the course of RT could arise both from anatomical variations and from clinical response (i.e., shrinkage and/or chemotherapy). Although the exact PTV in each stage of the treatment remains a controversial subject, we had chosen this particular contouring methodology because the idea of the study was to assess only dose variations arising from anatomic changes and not from a clinical response. This methodology was used previously by other authors.^(16)^

In order to eliminate differences in volumes due to interobserver variability, the three scans of each patient were contoured by the same physician.

The initial plan was calculated at the ${\text{CT}}_{15}$ and ${\text{CT}}_{25}$ and dose‐volume differences related to ${\text{CT}}_{\text{plan}}$ were assessed on PTVs and organs at risk.

### E. Statistical analysis

Comparisons of dose‐volume parameters were assessed among all three CTs. Correlation between volume changes and weight loss was evaluated. All dose‐volume parameters were reported following the ICRU 83 recommendations. The analyzed variables for GTV and PTV were: 1) volume, and 2) $D_{2\%}$, $D_{98\%}$, and $D_{50\%}$. For OARs, the studied variables were: 1) volume, and 2) $D_{2\%}$, $D_{\text{mean}}$, and 3) volume receiving absorbed dose $D(V_{D})$, where D is the absorbed dose which, if exceeded within some volume, has a high probability of causing a serious complication. D is specifically defined for each structure. A random effects model for repeated measurements was fitted to assess differences between the planning CT and later CTs. A value of p<0.05 was considered significant.

All analyses were performed with STATA 10.1 (StataCorp. 2009. Statistical Software: Release 10.1. College Station, TX).

## III. RESULTS

### A. Patient characteristics

Between November 2008 and March 2010, a total of 16 patients with bilateral neck and supraclavicular nodes treated with IMRT were included. Table 1 shows patient characteristics. Fourteen patients received radical treatment consisting of IMRT 70 Gy in 33 fractions of 2.12 Gy/fr, and systemic therapy specifically, Cisplatin for 13 of them ($100\;\text{mg}/m^{2}$; days 1, 22, 43) and a weekly administration of Cetuximab (anti‐EGFR) for the remaining patient. Two patients received postoperative IMRT with 64.5–66 Gy in 30–33 fractions of 2.15–2 Gy/fr, respectively.

**Table 1:** Patients' characteristics.

		*N*	*%*
Age	Mean (range)	16	58 (29–77) years
Gender	Male	10	62.5
	Female	6	37.5
Primary site	Nasopharynx	1	6.3
	Oropharynx	7	43.8
	Oral Cavity	5	31.3
	Hypopharynx	1	6.3
	Unknown	2	12.5
T Stage	Tx	2	12.5
	T1‐T2	5	31.3
	T3	4	25.0
	T4	5	31.3
N Stage	N0	3	18.8
	N1	2	12.5
	N2	10	62.5
	N3	1	6.3

### B. Volume and weight changes

The average relative weight loss with regard to the initial weight was statistically significant, with a reduction of 3% (p=<0.0009) and 4.5% (p=<0.0001) for ${\text{CT}}_{15}$ and ${\text{CT}}_{25}$, respectively. One of the patients showed a weight gain of 5.5% at ${\text{CT}}_{15}$ and 7% at ${\text{CT}}_{25}$. A new mask was required at ${\text{CT}}_{15}$ due to this increase.

Significant changes were found at the limited external volume, with an average decrease of 3% on both CTs (a range of: ‐16.3 to 12.9 for ${\text{CT}}_{15}$ and ‐16.4 to 11.1 for ${\text{CT}}_{25}$), with the maximum values corresponding to the sole patient with weight gain. Correlation between the weight change and the limited external volume variations was significant, with p=0.006 for ${\text{CT}}_{15}$ and p=0.001 for ${\text{CT}}_{25}$ with respect to the ${\text{CT}}_{\text{plan}}$.

One patient showed an important decrease in their limited external volume without weight loss (i.e., due exclusively to tumor shrinkage). Of the remaining patients, 10 (63%) exhibited weight loss which was more significant at ${\text{CT}}_{25}$ than at ${\text{CT}}_{15}$, and 4 (25%) suffered weight loss during the three first weeks, after which their weights remained stable.

Differences between the initial average volumes measured at ${\text{CT}}_{\text{plan}}$ and the two subsequent CTs were assessed for target volumes and OARs. For target structures, only PTV2 had a significant volume decrease of 13% (range: ‐58.2, 20.7) at the ${\text{CT}}_{25}$.

The parotid glands showed a progressive mean volume reduction of 22% at ${\text{CT}}_{15}$ and 30% at ${\text{CT}}_{25}$, corresponding to a mean reduction of 1.4% per treatment day (td). No other OAR showed a significant volume variation. Mean volumes, averaged over the 16 patients, and *p* values are shown on Table 2 for the PTVs and the OARs with statistically significant changes only.

**Table 2:** Volumes with significant changes on ${\text{CT}}_{15}$ ${\text{CT}}_{25}$ in relation to ${\text{CT}}_{\text{plan}}$.

	*PTV2*		*Limited External Contour*		*Contralateral Parotid Gland*		*Ipsilateral Parotid Gland*	
	*Mean* ^a^ *(SD)*	*p*	*Mean (SD)*	*p*	*Mean (SD)*	*p*	*Mean (SD)*	*p*
${\text{CT}}_{\text{plan}}$	130.8 (73.1)		1692 (412)		18.71 (10.3)	19.2 (10.6)	
${\text{CT}}_{15}$	116.7 (54.8)	0.07	1639 (402)	0.017	14.2 (6.6)	<0.001	15.3 (8.3)	0.003
${\text{CT}}_{25}$	113.6 (53.5)	0.03	1638 (415)	0.014	13.1 (6.4)	<0.001	13.5 (7.2)	<0.001

^a^ The mean value is the average volume between 16 patients, in ${\text{cm}}^{3}$.

SD is the standard deviation; p<0.05 is statistically significant.

### C. Dosimetric changes on target volumes

Table 3 shows target volume averaged dose parameters at ${\text{CT}}_{\text{plan}}$, ${\text{CT}}_{15}$, and ${\text{CT}}_{25}$. Values are presented as a percentage of $D_{\text{pres}}$ of each PTV. Average $D_{2\%}$ values increased in all target volumes, with the most significant increase seen at ${\text{CT}}_{25}$. In contrast, the dose covering the 95% and 98% of the volume decreased significantly at ${\text{CT}}_{15}$ for PTV2 and in both CTs for PTV1.

**Table 3:** Averaged dose‐volume parameters on ${\text{CT}}_{\text{plan}}$, ${\text{CT}}_{15}$, and ${\text{CT}}_{25}$ for target volumes.

*Target Volume*		$D_{2\%}$		$D_{50\%}$		$D_{95\%}$		$D_{98\%}$	
		*Mean* ^a^ *(SD)*	*p*	*Mean (SD)*	*p*	*Mean (SD)*	*p*	*Mean (SD)*	*p*
GTV	${\text{CT}}_{\text{plan}}$	111.4 (3)		107.1 (2.5)		103.7 (2)		103.0 (1.7)	
	${\text{CT}}_{15}$	112.6 (3.7)	0.145	107.7 (2.7)	0.094	103.3 (2.8)	0.491	101.2 (4.3)	0.158
	${\text{CT}}_{25}$	113.7 (4.7)	0.006	108.2 (2.7)	0.001	104.3 (2.5)	0.375	103.4 (2.5)	0.616
PTV2	${\text{CT}}_{\text{plan}}$	110.3 (3.1)		105.1 (1.7)		100 (0)		97.6 (1.3)	
	${\text{CT}}_{15}$	112.1 (4.6)	0.036	105.1 (2.7)	0.913	96.4 (7)	0.012	92.0 (9.3)	0.003
	${\text{CT}}_{25}$	112.6 (4.7)	0.008	105.8 (2.3)	0.109	98.9 (3.6)	0.414	95.8 (5)	0.348
PTV1	${\text{CT}}_{\text{plan}}$	127.0 (6.4)		111 (4.5)		101.2 (3)		98.2 (2.8)	
	${\text{CT}}_{15}$	130.0 (6.9)	<0.001	111.3 (5.7)	0.615	96.7 (6.7)	0.05	90.3 (11)	0.002
	${\text{CT}}_{25}$	130.8 (6.8)	<0.001	111.6 (5.5)	0.373	97.1 (7)	0.01	91.5 (11)	0.008

^a^ All values are averaged percentages of $D_{\text{press}}$ for each PTV and standard deviation (SD). $D_{V}$ absorbed dose that covers the fractional volume V. A value of p<0.05 is statistically significant. The prescribed volume was 95% of PTV2 so $D_{50\%}$ values are higher than $D_{95\%}$. For PTV1, $D_{50\%}$ and $D_{2\%}$ are high because of the influence of high dose areas close to PTV2.

### D. Dosimetric changes on organs at risk

Table 4 summarizes dose distribution changes on OAR, which showed some significant variation between planning CT and ${\text{CT}}_{15}$ and ${\text{CT}}_{25}$. All reported doses were percentages of $D_{\text{pres}}$. For the ipsilateral parotid gland, there was a $D_{m}$ and $V_{26\text{Gy}}$ significant increase at the ${\text{CT}}_{25}$ of 4.7% and 6.3%, respectively. For the contralateral parotid gland, there were significant ${\text{CT}}_{15}$ and ${\text{CT}}_{25}$ increases in the following: $D_{m}$ (3.6% and 6.1%); $D_{2\%}$ (5.9% and 7.7%), and in the $V_{26\text{Gy}}$ (7.4% and 10.4 %). For the spinal cord, the most notable dose increase was observed at ${\text{CT}}_{15}$, measuring a $D_{2\%}$ increase of 2.5% or 1.8 Gy.

**Table 4:** Organs at risk with statistically significant dose changes between planning CT, ${\text{CT}}_{15}$, and ${\text{CT}}_{25}$.

*Normal Structure*	$D_{2\%}$			$D_{mean}$		*V* ^b^_*D*_	
		*Mean* ^a^ *(SD)*	*p*	*Mean (SD)*	*p*	*Mean (SD)*	*p*
Ipsi Parotid	${\text{CT}}_{\text{plan}}$	89.7 (9.7)		44.5 (6.3)		49.7 (15.7)	
Gland	${\text{CT}}_{15}$	90.5 (12.5)	0.760	47.0 (10.9)	0.188	52.7 (20.3)	0.285
D=26Gy	${\text{CT}}_{25}$	93.8 (10.6)	0.102	49.2 (11.1)	0.012	56 (19.7)	0.024
Contra Parotid	${\text{CT}}_{\text{plan}}$	80.7 (11.3)		42.8 (5.2)		48.2 (10.4)	
Gland	${\text{CT}}_{15}$	85.5 (9.2)	0.045	46.4 (6.3)	0.051	55.6 (11)	0.041
D=26Gy	${\text{CT}}_{25}$	86.9 (12.8)	0.009	48.9 (8.2)	0.001	58.6 (14.4)	0.004
Mandible	${\text{CT}}_{\text{plan}}$	100.2 (5.8)		64.3 (5.7)		9.8 (8.9)	
D=66Gy	${\text{CT}}_{15}$	101 (5.8)	0.148	64.7 (6)	0.445	10.6 (9.3)	0.35
	${\text{CT}}_{25}$	101.9 (5.7)	0.003	64.9 (6.6)	0.293	11.2 (9.3)	0.091
Oral Cavity	${\text{CT}}_{\text{plan}}$	90.8 (10.1)		63.8 (14.1)		56.5 (25.1)	
D=40Gy	${\text{CT}}_{15}$	94.5 (9.7)	<0.001	67.3 (14.2)	0.001	62.3 (22.4)	0.004
	${\text{CT}}_{25}$	92.9 (10.3)	0.016	66.2 (14.7)	0.006	59.8 (25.4)	0.098
Middle low	${\text{CT}}_{\text{plan}}$	87.8 (10.6)		63.3 (8.2)		60.3 (24.3)	
neck	${\text{CT}}_{15}$	91.3 (12)	0.035	65.2 (8.1)	0.01	65.7 (22.7)	0.001
D=40Gy	${\text{CT}}_{25}$	89.7 (13.2)	0.252	64.9 (8.7)	0.031	64.0 (22.5)	0.024
Lips	${\text{CT}}_{\text{plan}}$	63.1 (16.8)		42.4 (11.4)		81.5 (16.5)	
D=20Gy	${\text{CT}}_{15}$	67.9 (17)	0.009	46.2 (13.4)	0.003	88.2 (15.8)	0.01
	${\text{CT}}_{25}$	68.4 (19.5)	0.003	48.4 (14.5)	<0.001	88.6 (13.5)	0.006
Spinal cord	${\text{CT}}_{\text{plan}}$	62.9 (3.5)				0.0 (0)	
D=48Gy	${\text{CT}}_{15}$	65.4 (7.2)	0.021			0.3 (1)	0.01
	${\text{CT}}_{25}$	64.8 (5.2)	0.091			0.2 (0.4)	0.013

^a^ The mean value is the average data between 16 patients and SD is the standard deviation. $D_{V}$ represents the absorbed dose that covers the fractional volume V, and is expressed as a % of the prescribed dose.

^b^ $V_{D}$ means percentage of total volume receiving a D dose. For spinal cord volume it is in cc. A value of p<0.05 is statistically significant.

Mean absorbed doses increased at ${\text{CT}}_{15}$ and ${\text{CT}}_{25}$ in the following structures: the lips (3.8%, 5.3%), the oral cavity (3.5%, 2.5%), and lower middle neck structures (1.9% and 1.6%). For lips, $V_{20\text{Gy}}$ increased in both CTs by 6.7% and 7.1%, respectively. $V_{40\text{Gy}}$ increased in ${\text{CT}}_{15}$ and ${\text{CT}}_{25}$ in the following organs: the oral cavity (5.7% and 3.3%), and lower middle neck structures (5.4% and 3.7%). No significant dose changes were found in the remaining OARs, namely the eyes, cochlea, brainstem, and brain.

No significant correlation was found between target and OAR dose increases with neither patient weight loss nor volume changes.

## IV. DISCUSSION

A limitation of our work is the difficulty to separate influences over dose distribution due to rigid errors (i.e., interfractional setup and intrafractional variations), and nonrigid anatomical changes during the course of the treatment. Additionally, our results have to be interpreted with a degree of caution as some of the dose variations with respect to the initial plan are small and can therefore be affected by other variables, such as calculation grid size and random co‐registration variability in the CTs.^(23)^ Finally, although statistical models attempt to reduce the impact of extreme variations, such as the patient who gained weight in this study, the sample size can significantly affect results, as any variation will have more influence in a smaller sample. Despite the limited number of patients entered in our study (16), an important part of our results were nevertheless statistically significant. Furthermore, our sample size is also larger than most used in published works related to this topic — for example, studies carried out by Barker et al.,^(8)^ Hansen et al.,^(16)^ Robar et al.,^(13)^ Lee et al.,^11^, ^15^ and Castadot et al.^(9)^ included 14, 13, 15, 10, and 10 patients, respectively. Taking into account all of the above factors, the average data shown in Tables 3and 4 could therefore help to identify dosimetric variables at risk of suffering changes during patient radiation treatment.

The study shows that HNC patients treated with IMRT undergo weight changes correlated with limited external volume variations, suggesting that patient weight may be a reliable parameter to detect changes in irradiated body areas. Weight loss is appreciable after the third week of RT treatment.

Despite attempts being made to maintain the original PTVs size, volume change was significant in PTV2 because, in many cases, volumes lying beyond the external contour had been modified.

The target coverage loss during the first part of the treatment, in which no significant target volume changes occurred, can be related to the anatomical changes observed (i.e., changes in the external volume). Our results coincide with Ahn et al.,^(18)^ who studied 23 HNC patients undergoing regular scans and found a loss of PTV coverage at approximately the 11th fraction, which was recovered in the posterior scans.

The parotid glands suffered a significant volume reduction and dose increase as treatment advanced. We show a significant averaged increase in $D_{\text{mean}}$ in the parotid glands of 5.4% (3.8 Gy) at the 25th fraction, consistent with Lee et al.,^(15)^ who reported a difference between planned and received mean doses of less than 10% in 70% of studied patients. In our study, the parotid glands suffered a mean reduction of 1.4%/td, consistent with 1.5%/td in the study by Bhide et al.,^(12)^ although a little higher than Castadot et al. and Lee et al.^9^, ^15^ of 0.9%‐1.0%/td. No correlation was found between volume reduction and dose. This may be due to one of many factors including a potential medial translation of the glands into high‐dose areas, as reported by several authors.^8^, ^9^, ^11^

None of the remaining normal tissues studied suffered volume variations. Nonetheless, the spinal cord and mandible showed a $D_{2\%}$ increase. The oral cavity, lips, and lower middle neck structures suffered a mean dose increase significant in the two CTs, indicating that body volume reduction inside the treatment area most likely contributed to a dose increase. Dose variation over these structures should therefore be considered.

For HNC patients treated with 3D CRT, except in the case of larynx and hypopharynx tumors, a central block is usually employed in the anterior neck field in order to avoid dysphasia and swallowing problems related with middle neck structure irradiation. In this study, lower middle neck structures were included because treating the entire neck with IMRT nonmatching fields decreases the risk of hot or cold spots due to errors in collimator positioning, while at the same time increases the dose in the lower middle neck.^(24)^ In our statistical data, the volume of middle neck structures receiving more than 40 Gy was significantly increased in both CTs with an average increment of 4.6%.

Dose deviations in relation to the initial dose in organs such as the oral cavity, lips, and middle lower neck have not yet been studied, and we think therefore that it is of interest to evaluate the effect of the above deviations on patients entered in our study. Table 5 shows the dose variation range of $D_{\text{mean}}$ and $V_{D}$ with respect to initial dose in the aforementioned structures in 16 of the patients. The number of patients with dose increments up to 10% is reported; the number in parenthesis indicates patients coinciding in the two CTs. The number of patients exceeding a 10% difference is larger in $V_{D}$ than in $D_{\text{mean}}$. In the majority of cases, patients with values above 10% in $V_{D}$ are not necessarily the same patients in whom $D_{\text{mean}}$ was increased. These specific OARs should be contoured and monitored in order to know if the initial plan remains suitable during the course of the treatment.

**Table 5:** Dose variation range of $D_{\text{mean}}$ and $V_{D}$, for the oral cavity, lips, and middle lower neck, and number of patients with dose increase up to 10%.

*Normal Structure*		$D_{mean}(\%)$		$V_{D}(\%)$ ^a^	
		*Range*	*Patients with dose increase up 10%*	*Range*	*Patients with dose increase up 10*
Oral Cavity	${\text{CT}}_{15}$	‐2.5, 6.2	0	‐7.4, 22.2	4
D=40Gy	${\text{CT}}_{25}$	‐2.4, 10.9	1	‐5.1, 21	2 (1)
Middle low neck	${\text{CT}}_{15}$	‐1, 9.6	0	‐1.8, 16.3	4
D=40Gy	${\text{CT}}_{25}$	‐2.3, 8.8	0	‐17.7, 19.3	3 (0)
Lips	${\text{CT}}_{15}$	‐3.4, 14.5	2	‐12.1, 29	5
D=20Gy	${\text{CT}}_{25}$	‐0.5, 14.9	3 (1)	‐2.5, 29	3 (3)

^a^ $V_{D}$ means percentage of total volume receiving a D dose.

To correct the effect of anatomical changes over dose distribution during the treatment, an adaptive radiotherapy (ART) approach is used. Schwartz and Dong^(25)^ have reviewed current investigations of ART in HNC, and conclude that ART remains an unsolved problem because the optimal frequency, utilization, and clinical impact of ART remain undefined. Moreover, advances in automated planning techniques based on deformable image registration will help to improve the feasibility of ART in HNC patients. ART and deformable image registration are still in development and are, therefore, not clinically implemented in the majority of hospitals.

Although replanning during the course of treatment is a time‐consuming process, it has potential advantages and is readily accessible for many centers. It should be stressed that it will be necessary to identify which patients in particular would benefit from replanning. For instance Ahn et al.^(18)^ indicated that only 15 out of 23 patients (65%) benefitted from a new plan. Recently, Zhao et al.^(26)^ showed that nasopharyngeal carcinoma patients who have clinically identified anatomic changes during the course of IMRT might potentially benefit from replanning.

For now, if ART rationale were used, the benefit of replanning would have to be analyzed individually. There are many issues which could determine the need for a replan: clinical patient conditions, and percentages of initial dose distribution variations for each PTV and OARs. The decision to consider suitable dose distribution is always a compromise between dose in target volumes and dose in OARs. Furthermore, both facilities and human/economical resources could determine the final decision for replanning. These complex factors are beyond the scope of this work but until now, no predictive factors that might identify the need for replanning have been reported. It is therefore essential to establish a rational adaptive RT scheme compliant with defined dosimetric criteria. The dosimetric criteria employed to select which patients could benefit most from replanning should be based on the knowledge of spatial dose variations on both target volumes and organs at risk during the course of treatment. Dose variations reported in our study may therefore be of some help for future investigations concerning adaptive radiotherapy.

## V. CONCLUSIONS

Variations in patient positioning and anatomical changes during IMRT for head‐and‐neck cancer can modify dosimetric parameters and may therefore have clinical implications on local control and toxicity. We have seen that the dose distribution over target volumes shows a decrease of coverage in the first part of the treatment and an overdosage towards the final part of the treatment. The mean dose in the parotid glands showed significant increases both for the contralateral than ipsilateral parotid gland. The present work has included structures related with new IMRT toxicities with the oral cavity, lips, brain, and lower middle neck irradiation. It is, to our knowledge, the first study analyzing the influence of volume changes over dose distribution on these particular organs at risk. Results have shown that there is a significant dose increase in the oral cavity, lips, and lower middle neck, although these structures did not present volume variations.

Behavior of dose distribution over target volumes and OARs depends on several aspects, namley particular volume changes, localization, and the irradiation technique employed. The IMRT technique implies the irradiation of a larger number of OARs than conventional 3D CRT, some of them at high doses. In conclusion, appropriate OARs have to be contoured and monitored in order to know if the initial plan remains suitable during the course of the treatment. Reported dosimetric data can help to identify patients who could benefit from adaptive radiotherapy
